# Integration of HIV pre-exposure prophylaxis (PrEP) services for pregnant and breastfeeding women in eight primary care clinics: results of an implementation science study

**DOI:** 10.1186/s44263-024-00089-8

**Published:** 2024-08-26

**Authors:** Aurelie Nelson, Kalisha Bheemraj, Sarah Schoetz Dean, Alex de Voux, Lerato Hlatshwayo, Rufaro Mvududu, Natacha Berkowitz, Caroline Neumuller, Shahida Jacobs, Stephanie Fourie, Thomas Coates, Linda Gail-Bekker, Landon Myer, Dvora Joseph Davey

**Affiliations:** 1 Division of Epidemiology & Biostatistics, School of Public Health, University of Cape Town, Cape Town, South Africa; 2 Division of Infectious Diseases, Geffen School of Medicine, University of California Los Angeles, Los Angeles, USA; 3 City of Cape Town Department of Health, Cape Town, South Africa; 4 Western Cape Department of Health and Wellness, Metro Health Services, Klipfontein Mitchell’s Plain Sub-Structure, Cape Town, South Africa; 5 Desmond Tutu Health Foundation, Cape Town, South Africa

**Keywords:** Pre-exposure prophylaxis, HIV, Maternal and child health, Implementation science, Vertical transmission

## Abstract

**Background:**

Although HIV vertical transmission has declined significantly in sub-Saharan Africa, incident HIV infection in pregnant and postpartum women is estimated to account for over one-third of HIV vertical transmission. Oral pre-exposure prophylaxis (PrEP) for pregnant and breastfeeding women (PBFW) is included in South African PrEP guidelines since 2021; however, integration of PrEP services within ante- and postnatal care remains limited.

**Methods:**

Between March 2022 and September 2023, we evaluated the integration of PrEP for PBFW in eight antenatal clinics in Cape Town, South Africa, following training and mentorship of providers. We applied an adapted Reach, Effectiveness, Adoption, Implementation, and Maintenance (RE-AIM) framework to evaluate the integration of PrEP services for pregnant and breastfeeding women. Before the study, PrEP was not routinely offered. We implemented a staff didactic/practice-based training and mentorship on PrEP provision targeting PBFW. We evaluated the following: (1) Reach as the proportion of women initiating PrEP among women counselled and tested for HIV, (2) effectiveness as PrEP continuation up to 3 months by pregnant vs. breastfeeding women, (3) adoption of PrEP integration via pre- and post-training assessments and ongoing mentorship assessments, (4) implementation through clinic trends of PrEP offer over time, and (5) maintenance: continued PrEP offer 3 months following the intervention.

**Results:**

In 8 facilities providing ante- and postnatal care, we trained 224 healthcare providers (127 nurses and 37 counsellors). Of those, we mentored 60 nurses, midwives, and HIV counsellors working with pregnant and breastfeeding women, with 80% of nurse/midwives and 65% of counsellors scoring ≥ 80% on the final mentoring assessment. Overall, 12% of HIV-negative pregnant women started PrEP, and 41% of those continued PrEP up to 3 months. Among HIV-negative breastfeeding women, 14% initiated PrEP, and 25% continued PrEP up to 3 months. All eight facilities continued providing PrEP 3 months post intervention.

**Conclusions:**

In these high HIV prevalence clinics, the proportion of pregnant and breastfeeding women initiating and continuing PrEP rapidly increased but was limited among breastfeeding women. Staff training, mentorship, and PrEP integration were well-adopted by nurses and counsellors, and services continued following the intervention. Barriers included limited HIV testing of breastfeeding mothers and need for additional PrEP-trained nurses.

**Supplementary Information:**

The online version contains supplementary material available at 10.1186/s44263-024-00089-8.

## Background

Vertical HIV transmission has declined significantly in sub-Saharan Africa in the last decade, with an estimated 76% reduction in South Africa, thanks to effective interventions such as the roll out of antiretrovirals (ARVs) to pregnant women living with HIV regardless of their CD4 counts [1–3]. Despite this, South Africa still has one of the highest rates of vertical transmission globally, estimated at 3% in 2022, resulting in a predicted 90,000 new paediatric infections over the next 10 years [2, 4, 5]. Over one-third of HIV vertical transmission can be attributed to incident HIV infection in mothers whilst pregnant or breastfeeding [5]. It is estimated that pregnant and breastfeeding women (PBFW) are 3.6 times more likely to contract HIV than nonpregnant women, a recent prospective study identifying the postpartum period as the highest risk time for HIV acquisition [6, 7]. These data reinforce the need for improved primary HIV prevention among PBFW not living with HIV to achieve elimination of maternal HIV acquisition and vertical transmission of HIV.

Although prevention of HIV is multipronged, one of the few effective methods that the female partner can use without male partner use is the use of daily oral tenofovir-disoproxil-fumarate/emtricitabine-based (TDF/FTC) pre-exposure prophylaxis (PrEP) [8, 9]. A recent systematic review including 14 studies found TDF/FTC exposure had no impact on pregnancy or perinatal outcomes [10, 11]. Furthermore, very little TDF/FTC is excreted in breastmilk [12]. In South Africa, modelling the impact of PrEP provision to PBFW shows between 48,000 and 136,000 averted new HIV infections among PBFW over the next 10 years, resulting in a significant decrease in vertical transmission between 13 and 41% [4]. Based on this evidence, the World Health Organization (WHO) recognised that the benefit of taking PrEP for PBFW to prevent incident infections and vertical transmission outweighs the potential risks for mother and child [13]. In October 2021, the South African Department of Health (DOH) changed the PrEP guidelines to include provision of PrEP to PBFW at risk of HIV acquisition [14].

Studies in other sub-Saharan African contexts have demonstrated feasibility of PrEP implementation in maternal and child health (MCH) clinic settings [15]. However, recent South African studies have shown that PBFW face several barriers to accessing PrEP, such as anticipated and internalised stigma and challenges in disclosure of PrEP use [16, 17]. Studies conducted across several different contexts have found that many healthcare providers (HCPs) describe their PrEP knowledge as insufficient, resulting in inaccurate patient education and HIV counselling, hesitancy to prescribe PrEP, and a lack of confidence in completing PrEP-related clinical activities [18, 19]. Furthermore, studies conducted in sub-Saharan Africa found that many providers believed that oral PrEP may be inappropriate during pregnancy and for adolescent girls due to negative beliefs about adolescent sexuality and doubts that young women could manage the daily pill burden [19, 20]. PrEP training among counsellors and healthcare providers has been shown to improve uptake and access to HIV counselling and testing, as well as PrEP screening, uptake, and adherence [12, 18, 21, 22]. Further research suggests that integration of PrEP into antenatal and postnatal care settings can enhance PrEP initiation and continuation among PBFW [15, 21–23]. Together, these findings highlight the importance of practical provider PrEP training for the effective integration of PrEP into routine care for PBFW.

However, despite these findings, there is a gap in the delivery of strategies and applications to real-world conditions [24]. The implementation science approach chosen for this study addresses this gap by applying the Reach Effectiveness-Adoption Implementation Maintenance (RE-AIM) framework to evaluate the integration of PrEP services into ante- and postnatal care services following training and mentorship of healthcare providers (HCPs; nurses and HIV counsellors) in eight clinics with high HIV prevalence within Cape Town, South Africa [25].

## Methods

### Study design and setting

We aimed to systematically evaluate integration of PrEP services for PBFW in eight primary care clinics in the Klipfontein and Mitchells Plain subdistricts, South Africa, between March 2022 and July 2023, with continued post-intervention monitoring through September 2023. Selected clinics were in Cape Town high-density areas, with antenatal HIV prevalence ranged from 10 to 35% and were selected in consultation with the local district Department of Health based on client volume, geographical location, and HIV prevalence [26]. Community health centres (CHC) included larger clinics which include 24-h services and midwifery obstetric units (MOUs), defined as large antenatal facilities where women were referred to from smaller antenatal care clinics after 36 weeks of gestation to deliver. Community day centres (CDC) did not have a 24-h unit and provide basic antenatal clinic services as well as baby wellness services, among other services.

### Implementation strategy

**Fig. 1:**
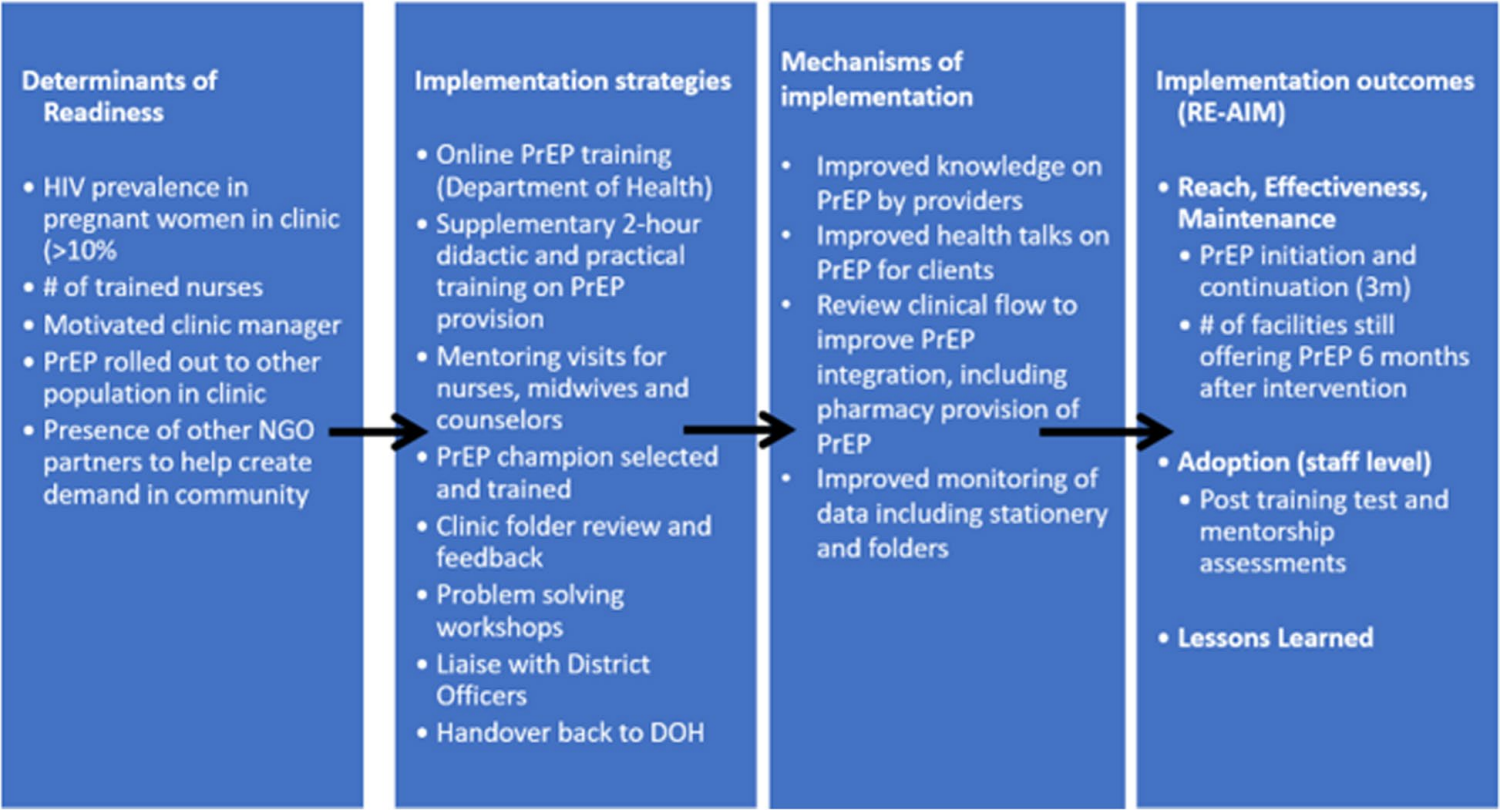
Description of the oral pre-exposure prophylaxis (PrEP) in pregnant and breastfeeding women (PBFW) training and mentorship intervention from determinants to outcome

The implementation study staff was comprised of two research nurses, two counsellors, one research assistant, and one medical doctor with experience implementing PrEP services among PBFW within the region. First, the team assessed the readiness of the facility by assessing the following: (1) the antenatal HIV prevalence (health need); (2) the number of ART-trained nurses available for providing PrEP (provider availability); (3) verbal confirmation of the facility manager to want to extend the offer of PrEP to PBFW (management motivation); (4) whether the facility had rolled out PrEP for other populations (clinic capacity); (5) confirmed presence of non-governmental organizations (NGOs) in the clinic to assist with demand creation for PrEP (via health talks in the clinic, community promotions and other information, education, and communication campaigns); and (6) PrEP availability at the facility. If the clinic was not ready per abovementioned criteria, we aimed to address existing structural issues with the local clinical governance officers and revisit the facility 3 months later. Following the readiness assessment, the clinic manager identified the team to train and a date for training and determined a designated PrEP champion at the clinic. The PrEP champion was responsible for leading the implementation of PrEP at the clinic level, such as troubleshooting, reporting any PrEP-related issues to the implementation team, and following up on training (Fig. 1).

### Implementation training

The implementation team advised the clinic team to complete the online DOH training prior to their training, and then they trained HCPs (e.g. managers, midwives, nurses, counsellors, and clerks) during a 2-h didactic session, including a practical session with nurses and counsellors [27]. Trainees completed a pre-training assessment, consisting of 10 questions on their knowledge about PrEP safety and effectiveness, HIV prevention in pregnancy, and completing a demographic questionnaire on their age, gender, and previous trainings. After a brief introduction to PrEP, the team was divided into clinician and counselling groups for practical training. The clinical team received DOH-based PrEP training by the study nurse on PrEP initiation and continuation. In parallel, the study counsellor led a practical session for the counsellors on PrEP and daily adherence support. The clinical and counselling teams then regrouped to discuss any potential barriers in the clinic flow and found solutions together on how to address them. A post-training test was then completed by all trainees and assessed by the study doctor/nurse, with a passing score of 80% or higher. If a provider scored < 80%, the training information was reviewed individually between the HCP and the study team to ascertain if the provider or counsellor was ready to start PrEP counselling and provision. The clinic team was also given educational posters and job aid materials regarding PrEP in PBFW for the HCP, developed by DOH, as well as pamphlets on PrEP in PBFW, in English and isiXhosa, designed, developed, and pretested by the study team.

### Implementation mentorship

The implementation team initially provided weekly mentoring to each clinic, wherein the study nurse and study counsellor only mentored trained nurses/midwives and counsellors who were working directly with PBFW. At each individual mentoring session (with 2–4 sessions occurring per site visit), the implementation team completed a quantitative assessment checklist from 0 to 10 items from the training and national guidelines. Trainees were assessed on their ability to counsel on (counsellors) or offer PrEP (providers) for HIV-negative women, regardless of a woman’s interest in PrEP (see Additional File 1) [28]. The scores ranged from 0 (poor) to 10 (excellent), with feedback and comments provided at each assessment. We set the score of 8 out of 10 as a successful application of the training, in discussion with DOH supervisors. This assessment allowed for measurement of PrEP integration by each provider within the clinic, defined as the degree to which the providers delivered PrEP counselling, initiation, and recording according to their training. As the mentored trainees progressed, the clinic mentoring frequency decreased to once per month. Each trainee received 2–5 mentoring visits in total, spanning the course of approximately 6 months, depending on operational needs.

In collaboration with the district clinical governance officers, we audited each participating clinic twice throughout the study, reviewing 10 folders of PBFW per clinic to review quality of documentation and PrEP prescription. Results of the file review were communicated to the nurses and midwifes involved, as well as to their respective managers, and actions were taken by the clinical DOH staff to correct any errors, inconsistencies, or missing data identified by the study team.

Six months after the initial training, the study team concluded mentorship visits. The study team met with the facility manager and trained providers to provide a report that included the data collected on offer, initiation, and continuation of PrEP among PBFW, results of the clinical folder reviews, and results of problem-solving discussion with the staff on barriers encountered. Following the end of mentoring, the study team continued collecting data on PrEP initiation and continuation in PBFW for at least 3 months.

### Enrolment criteria

Facility managers, counsellors, pharmacists, nurses, and midwives who were offered DOH and supplementary University of Cape Town (UCT) training and consented to their data being analysed were included in the study [27]. Per national guidelines, only nurses to prescribe antiretroviral therapy (ART) are allowed to prescribe PrEP. Provider data was collected from pre- and post-training questionnaires, including socio-demographic data (e.g. training, age, and gender). Providers in a role where they could counsel or provide PrEP to PBFW were mentored by experienced study trainer/mentors on a regular basis (initially weekly then monthly, ranging from 2 to 5 times), and corresponding data was collected using assessment checklists (see Additional file 1).

Following training and mentorship of HCPs in both antenatal care (ANC) and “baby wellness” clinics, we monitored the offer, initiation, and continuation of PrEP to eligible PBFW attending the clinic for their regular visits. Inclusion criteria included women eligible for PrEP as per the DOH PrEP guidelines and who did not opt out of aggregate data collection [29]. In facilities, study staff collected de-identified aggregate data from HIV-negative PBFW eligible for PrEP. Providers at the selected facilities who participated in the PrEP training were asked to provide written informed consent to have data from their pre- and post-training questionnaires, demographic information, and mentoring assessment collected. Passive consent was obtained at the clinics from women attending antenatal care and well-baby or immunisation visits with posters explaining the ongoing aggregate data collection about PrEP use and giving the option to women to opt out or withdraw consent at any time. All data was de-identified using de-linked patient identifiers. No reimbursement was provided for participating women nor providers in this implementation science study.

### RE-AIM framework

We used the RE-AIM framework to evaluate the integration of PrEP into ante- and postnatal care over 6 months [24, 25]. The application of the RE-AIM framework is summarised in Table 1.

- Reach: We determined the number of clinical encounters and individuals seen for antenatal and postnatal care during the study by reviewing the clinic routine data including the following: (1) the clinic attendance record, (2) HIV testing register (to identify number of women without HIV), and (3) clinic PrEP register (for initiation and continuation on PrEP) in each clinic.
- Effectiveness: We evaluated the proportion of patients who initiated and continued PrEP at 3 months by pregnant vs. postpartum/breastfeeding in each clinic by reviewing PrEP clinic registers. We also report on the PrEP cascade in terms of people tested and counselled for HIV, PrEP initiation, and continuation by clinic.
- Adoption: We determined the adherence to the planned training schedule and delivery of PrEP. We evaluated the proportion of staff and providers who attended planned training through attendance logs as well as their pre- and post-training tests and proportion who passed with ≥ 8/10 questions correct. We also report on in-session mentorship assessments in which the study staff assessed trained providers on 10 competencies, and 8 out of 10 was considered competent, in line with DOH expectations and training.
- Implementation: The programme goal was to offer universal HIV testing and offer of PrEP to all women. We report on trends in patient counselling and screening for PrEP through a review of the implementation log and PrEP data collected from clinic and staff.
- Maintenance: We evaluated adherence to the planned integration beyond the study period through a review of PrEP data in each clinic by pregnant and postpartum/breastfeeding clients in each clinic 3 months post end of intervention (approximately 6 months following the start of the intervention).

**Table 1:** Study outcomes mapped using the RE-AIM framework

**Construct**	**Definition**	**Outcomes**	**Measures**	**Data source**	**Frequency**
Reach	Individual-level measure of participation	Proportion and characteristics of women counselled on HIV prevention and initiated on PrEP	Number of patients served Proportion starting on PrEP by pregnancy vs. postpartum/breastfeeding	Clinic routine data: Counsellor’s HIV testing register, clinic attendance record, PrEP register	Completed by counsellor and nurses and captured weekly by research assistant
Effectiveness	Achievement of programme goals and objectives	PrEP continuation rate	Proportion of women who continue PrEP at up to 3 months by pregnant vs. postpartum/breastfeeding PrEP cascade from testing, initiation to continuation	PrEP Department of Health register PrEP register	PrEP register completed by providers during the refill visit. Captured weekly by research assistant
Adoption	Assessment of the delivery setting	Proportion of staff who adhered to training and PrEP delivery by staff cadre and setting	Proportion of staff and providers who participated in the training and intervention Barriers and facilitators to adoption (*to be reported in qualitative analysis in subsequent report*)	Pre- and post-training test PrEP mentoring assessment checklist — completed by the study nurse/counsellor	Before and after training During mentorship (over 6 months, following training)
Implementation	Extent to which the intervention was delivered	Adherence to the planned intervention	Trends in patient counselling and screening for PrEP	Implementation log and PrEP data collected from clinic and staff	Monthly data reviewed (spanning from training to end of mentorship)
Maintenance	Institutionalisation of the programme	Adherence to the planned integration beyond the study period	Current data from clinic on PrEP counselling and provision (following intervention)	Clinic routine data: Counsellor’s HIV testing book, PMTCT register, PrEP registers, and review of electronic data	PrEP initiation and continuation data among PBFW by clinic 3 months post end of intervention

*PrEP* pre-exposure prophylaxis, *PMTCT* prevention of mother to child HIV transmission

### Data collection

Information on reach and effectiveness was collected from two sources: (1) aggregate data from facility logs on all PBFW attending regular ANC and “baby wellness” visits and (2) patient logs kept by HCPs on prescribing and counselling clients on PrEP. Information for antenatal pregnant women and for breastfeeding women was collected separately. The number of ANC visits gives an indication of the headcount and size of each clinic. Because the number of breastfeeding women attending “baby wellness” clinics was not recorded, we used the number of babies attending the visits as a proxy. The number of clinic attendees, HIV tests conducted, HIV-negative test results, and women initiating and continuing PrEP was captured on a weekly basis by the study research assistant using standard clinic records and PrEP register. HIV antenatal prevalence was calculated by clinic and based on antenatal attendance and HIV prevalence at each clinic for the year 2021, as per the information supplied by the provincial DOH. Accuracy of the data collected was triangulated for the smaller City of Cape Town clinics by reviewing the clinic data on the monthly number of women attending ANC, having an HIV test with accompanying test results and initiating PrEP. Data codebook and data from study are in Additional Files 2 and 3.

### Statistical analysis

Reach and effectiveness metrics were calculated as the proportion of HIV-negative pregnant or breastfeeding women who initiated PrEP and continued to 3-month post-initiation, stratified by pregnant women vs. postpartum/breastfeeding women. Number of breastfeeding women was estimated from the number of immunisation visits as a proxy. The number of PrEP initiations were plotted separately for pregnant women in and breastfeeding women during mentorship and post-mentorship periods. A loess curve was plotted for all MOUs/clinics and CHC/CDCs to fit a smooth curve across the data points. Mean PrEP initiations during clinic mentorship vs. after were reported with proportion changes within pregnant and breastfeeding groups. Demographics and pre-training and post-training scores of HCPs were reported overall and by HCP groups using descriptive statistics. *T*-tests were used to compare proportion initiating in high and low HIV prevalence facilities. All analyses were done in R Project, v.4.3.3.

## Results

### Reach

There were 12,614 eligible HIV-negative pregnant women encountered during the study, and 1493 women initiated PrEP during pregnancy (12%). The clinics that had lower HIV antenatal prevalence (< 15%) had lower proportion of women who started PrEP with 251 women starting PrEP among 8100 eligible (3%, 95% *CI* = 2.7–3.5%). In the two clinics and one MOU that had a higher HIV antenatal prevalence (> 30%), PrEP initiation was significantly higher (mean difference = 26.6; *t*-test = 133.3; *p*-value: < 0.001) with 1081 of 3639 women initiating PrEP (30%, 95% *CI* = 28–31%). August 2023 had the greatest number of PrEP initiations (*n* = 100 of 980, 10%) among pregnant women in MOUs, whilst December 2022 had the lowest (*n* = 30 of 980, 3%). Across the five clinics, July 2023 had the highest PrEP initiations (*n* = 43 of 513, 8%) with the lowest in December 2022 (*n* = 9 of 513, 2%).

Among breastfeeding women, we observed 34,230 immunisation, or well-baby visits (proxy for postpartum visit) were conducted during the study. Overall, 1317 women tested HIV negative, and 179 initiated PrEP (14% initiation). Across the eight clinics, the proportion of breastfeeding women who initiated PrEP ranged from 2 to 28%, with higher proportion initiating in the clinics with higher HIV antenatal prevalence.

### Effectiveness

**Fig. 2:**
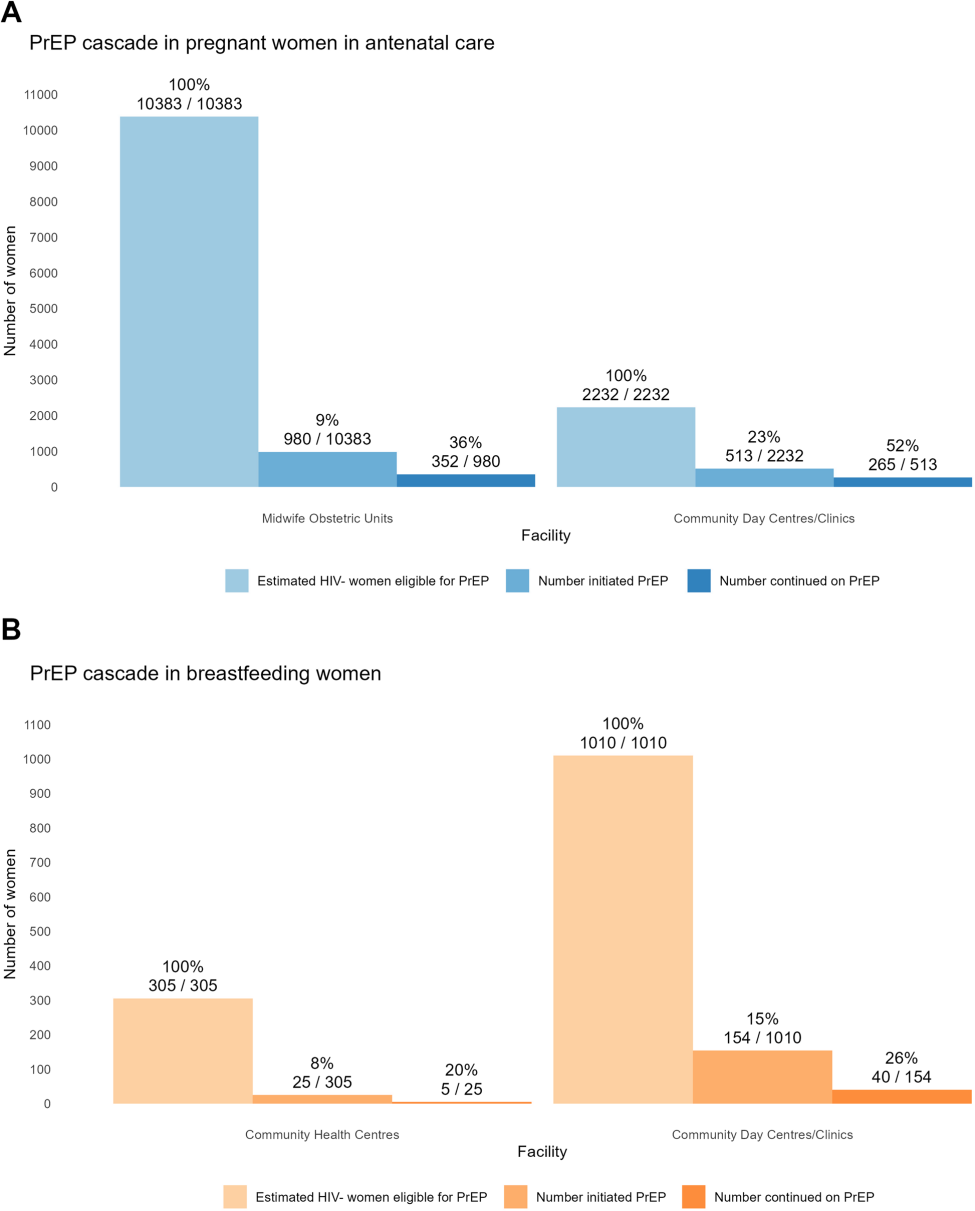
PrEP initiation and continuation cascade in **a** pregnant women in antenatal care and **b** breastfeeding women in eight clinics in Cape Town, South Africa (March 2022–September 2023)

The proportion of women who continued PrEP (measured as PrEP refill at up to 3 months) ranged from 23 to 70% by clinic, with an overall continuation proportion of 41% (617/1493) among pregnant women in all clinics. The proportion who initiated PrEP was greater in the MOUs (15%) vs. the clinic visits (8%, *p* < 0.001). Of those who initiated PrEP whilst breastfeeding, 45 (25%) continued PrEP up to 3 months. PrEP continuation among breastfeeding women at 3 months ranged from 0 to 59% by clinic (Table 2 and Fig. 2 for cascade by pregnant vs. breastfeeding women).

**Table 2:** Reach and effectiveness in pregnant and breastfeeding women without HIV and PrEP initiation and continuation in *n* = 8 antenatal care or baby-wellness facilities (March 2022–September 2023)

**Clinic**	**HIV antenatal prevalence (2021)**	**No. of ANC visits in pregnancy or no. of immunisation visits**	**No. of HIV tests in pregnant or breastfeeding women**	**No. of HIV-negative tests in pregnant or breastfeeding women**	**Estimated no. of HIV-negative pregnant/breastfeeding women eligible for PrEP**^a^	**Reach:** **No. and % PrEP initiated (of HIV negative tested)**	**Effectiveness:** **No. and % continued up to 3 m**
**Pregnant women in antenatal care**							
MOU (*n* = 3)	19%	64,091	16,843	16,612	10,383	980 (9%)	352 (36%)
Clinics/CDC (*n* = 5)	32%	5661	3623	3571	2232	513 (23%)	265 (52%)
**Total pregnant women**		**69,752**	**20,466**	**20,183**	**12,615**	**1493 (12%)**	**617 (41%)**
**Postpartum and breastfeeding women in infant immunisation or well-baby visits by clinic/CDC vs. CHC**							
CHC (*n* = 3)	19%	1260		306	305	25 (8%)	5 (20%)
Clinics/CDC (*n* = 5)	32%	33,060		1011	1010	154 (15%)	40 (26%)
**Total breastfeeding women**		**34,320**		**1317**	**1315**^b^	**179 (14%)**	**45 (25%)**

According to the data collected from City of Cape Town clinics, pregnant women tested for HIV on average 1.6 times per pregnancy in 2022, compared to three times as recommended in the DOH guidelines [30].

*MOU *midwife obstetric unit, *CHC *community health centre *CDC *community day centre *PrEP *pre-exposure prophylaxis

^a^Number of breastfeeding women estimated from the number of immunisation visits as a proxy.

^b^Two women subsequently tested HIV positive and were ineligible for PrEP start.

### Adoption

We evaluated the adoption of PrEP guidelines and training by HCP after reviewing post-training test questionnaire results and mentoring scores (see Additional File 3). Median pre-test score was 70% (*IQR*: 50, 80), and post-training score was 80% (*IQR*: 70, 90). Nurses and midwives’ median posttest scores were 80% for non-ART-trained cadres (*IQR*: 60, 80) and 90% for those ART trained (*IQR*: 80, 90). Counsellors’ pre-test median score was 60% (*IQR*: 50, 70), and posttest median score was 80% (*IQR*: 70, 80). Overall, 129 (58%) of HCPs had a post-training test score above 80%. The overall test results improved by 31% following training, with health assistant scores improving by 61% and counsellors by 34%.

For in-session mentorship assessment, median score was 8 out of 10 items evaluated (*IQR*: 7, 8.6), which did not improve over time (end mentorship score: 8, *IQR*: 8, 9). The proportion of trained, mentored staff with final mentorship score of ≥ 8 was 75% (45 of 60 staff), increased from 57% at baseline (34 of 60 staff). Counsellors had the lowest proportion achievement of the 8 of 10 on the mentorship assessment, with 13 of 20 (65%) achieving this score. Overall, 80% of nurses achieved the 8 out of 10 or greater on the mentorship assessment. Counsellors struggled to integrate the importance of disclosure of their PrEP use to others and how to establish a plan to take PrEP daily. For nurses, the most common area of improvement included reminder of PrEP refill appointment dates (Table 1 and Additional file 4).

### Implementation

**Fig. 3:**
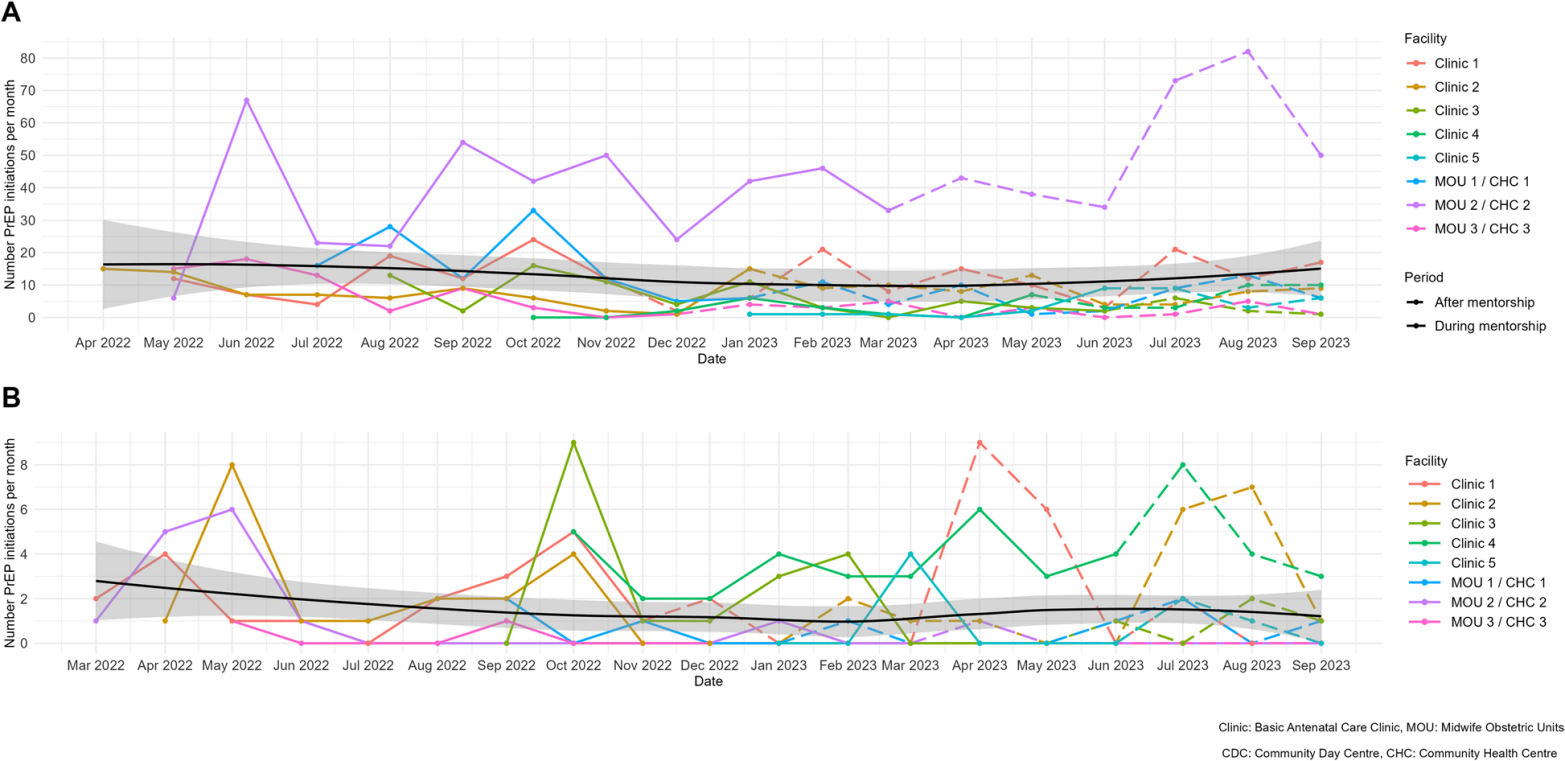
PrEP initiation in pregnant and breastfeeding women over time in Cape Town, South Africa, among the following: **a** Pregnant women initiating PrEP during mentorship and after mentorship and **b** breastfeeding women initiating PrEP during mentorship and after mentorship, with overall trend line across all clinics. CHC, community health centre; CDC, community day centre; MOU, midwife obstetric unit

Monthly clinical data and implementation logs revealed HIV testing occurring at 29% (20,466/69,752) of all ANC visits recorded during the period of observation. Among baby wellness and immunisation visits recorded, 4% included maternal HIV testing in which the mother was confirmed HIV negative (Table 2). Trends in monthly PrEP initiations are shown in Fig. 3 by pregnant women in ANC (Fig. 3a) and postpartum/breastfeeding women (Fig. 3b). In pregnant women, one of the clinics was an outlier with consistently high levels (> 50 pregnant women per month) of PrEP initiation, which improved after the government announced the PrEP in ANC indicator. Other clinics remained stable at approximately 10–20 PrEP initiations per month. In breastfeeding women, most clinics saw < 10 women initiating PrEP per month, with increases around mentorship periods (May 2022 and October 2022) and in the maintenance phase after the PrEP in ANC indicator was released.

### Maintenance

All eight clinics and MOUs provided PrEP 3 months following completion of training and mentorship. In Table 3, we describe in greater detail clinic-specific events during mentorship and following facility handover. For pregnant women, there was an overall decrease in the number of monthly average PrEP initiations by 8% following the intervention end. However, four clinics showed an increase in the number of monthly average PrEP initiations. Three of the four clinics which showed a decrease in monthly average PrEP initiations had lower HIV antenatal prevalence (< 30%) than the others. For breastfeeding women, the overall number of monthly average PrEP initiations mirrored that observed among pregnant women, decreasing by 21% following the intervention. Similarly, two clinics showed an increase in their average monthly PrEP initiation among breastfeeding women following the intervention, and three clinics had no PrEP initiations following the intervention.

**Table 3:** Maintenance of prescribing PrEP at the clinic level comparing the mean PrEP prescriptions whilst clinic was mentored to after the clinic was handed over

				**Pregnancy**			**Breastfeeding**		
**Clinic**	**Dates of staff mentoring**	**Months mentored**	**Antenatal HIV prevalence**	**%** change*	**1. Average PrEP initiations per month during mentorship**	**2. Average monthly PrEP initiations 3 months after handover**	% change*	**3. Average PrEP initiations per month during mentorship**	**4. Average monthly PrEP initiations in 3 months after handover**
Clinic/CDC 3	Aug 2022 Jun 2023	10	38%	53%↓	6	3	50%↓	2	1
Clinic/CDC 2	Apr 2022–Jan 2023	9	35%	10%↑	8	9	30%↓	2	1
Clinic/CDC 4	Sep 2022–Jun 2023	9	33%	118%↑	2	5	41%↑	4	5
MOU/ CHC 3	Apr 2022–Dec 2022	8	10%	48% ↓	8	4	No change	0	0
MOU/ CHC 2	Aug 2022–Mar 2023	7	34%	3%↑	37	38	69%↑	1	0
Clinic/CDC 1	Apr 2022–Nov 2022	7	28%	25%↓	13	10	68%↓	2	1
Clinic/CDC 5	Nov 2022–Jun 2023	7	24%	157%↑	2	6	No change	1	1
MOU/CHC 1	Jun 2022–Jan 2023	6	13%	65%↓	8	3	44%↓	1	0
**Total**				**8%**↓	**85**	**78**	**21%**↓	**13**	**9**

^*^Represents % change in number of monthly average PrEP initiations between the time the clinics were mentored and after the study mentorship ended.

*MOU *midwife obstetric unit, *CHC *community health centre (both have maternity facilities), *CDC *community day centre

## Discussion

In these high HIV prevalence clinics in Cape Town, South Africa, the proportion of PBFW initiating and continuing PrEP rapidly increased but was limited among breastfeeding women. PrEP uptake and continuation, however, were lower than expected compared to our recent cohort study, PrEP in pregnancy and postpartum (PrEP-PP) implemented in a similar setting using study staff, where over three-quarters of pregnant women without HIV enrolled in the study initiated PrEP and about half continued at 3 months [11]. Nurses and midwives demonstrated competency during ongoing mentorship assessments, but was lower among HIV counsellors, indicating the need for continued training and support. All eight facilities continued providing PrEP after the intervention, though with an overall decrease in PrEP initiation among PBFW over time. As PrEP initiation in this real-world setting fell below that of levels observed in related trial settings, further research is needed to understand unique experiences and perspectives among PBFW declining the offer of PrEP.

Clinics in higher HIV prevalence communities (> 20% antenatal prevalence) had higher levels of PrEP uptake and continuation compared to clinics in lower HIV prevalence communities among PBFW. This could be due to a variety of factors, including different risk perception and HIV-related stigma [31]. Surges in initiations and testing could be attributed to the pre-festive season with decreased proportion of HIV testing and PrEP initiation during the festive season, as documented elsewhere in South Africa [32]. This implementation science study was conducted in a real-world setting with no dedicated study staff providing counselling, PrEP prescriptions, or financial compensation for PBFW, as would be provided in a randomised controlled trial design. As such, our results are similar to a real-world implementation programme conducted in Kenya where PrEP delivery was integrated into 16 antenatal clinics and 18.6% of pregnant women were initiated on PrEP (of *n* = 4912 tested HIV negative) [23]. In a study of antenatal PrEP in Kenya, dedicated programme nurses offered PrEP counselling, whereas in our study, public sector staff were trained and mentored as the only individuals counselling and prescribing PrEP throughout.

Reasons for our comparatively lower PrEP uptake and continuation among PBFW could include stigma faced in certain communities, low-risk perception from women at substantial risk (for example with a partner of unknown HIV status), concerns around safety of PrEP in pregnancy, and sexual activity declining in late pregnancy compared to early pregnancy [23, 24, 33, 34]. Possible health-system barriers described in the literature include the additional work burden from PrEP-specific activities, PrEP hesitancy linked to inadequate HCP training, inappropriate PrEP beliefs stemming from lack of PrEP knowledge in HCPs, concerns around PrEP safety in pregnancy, and the need for specific monitoring of PrEP initiation in PBFW [19, 35, 36]. Some of the health system barriers observed on the ground, explored within forthcoming complimentary qualitative research on PrEP integration from the HCP point of view, were shortage and high turnover of staff and requirements for nurses prescribing PrEP to be ART trained [37]. ART-trained nurses are themselves in low numbers, overburdened, and not necessarily in services where PrEP can be integrated with other services [38].

Further, we identified that there was limited integration of maternal and child health, apart from the 6-week immunisation visit when testing of HIV-negative breastfeeding mothers occurred. The 3-monthly HIV testing among breastfeeding women was limited in most of the mentored clinics, with very little subsequent maternal and child integration. Despite postnatal transmission being a significant driver of vertical transmission of HIV, testing of breastfeeding women remains neglected in South Africa [7, 39, 40]. Therefore, in our study, the denominator of breastfeeding women testing for HIV was low at < 2000 women and only 179 (14%) of which started PrEP. In comparison, within the Kenyan study, 25% of postpartum women initiated PrEP of *n* = 4467 women testing HIV negative [23]. Possible reasons for the lower HIV testing include limited integration of maternal and child services, despite international and national recommendations [35, 41, 42]. However, after workshopping potential solutions to the identified barriers with clinic managers and staff, the number of PrEP initiations increased among breastfeeding women. With the release of new vertical transmission prevention guidelines calling for integration of maternal and child services postpartum, we expect that fixed testing timepoints for breastfeeding women (10 weeks, 6 months, and then quarterly) will increase HIV testing and the subsequent offer and counselling about the benefits of PrEP use in this period [41].

Continuation was slightly higher among pregnant women than in breastfeeding women and was comparative to the PrIYA study participants at the 3-month period [23, 35]. Lower persistence on PrEP could be partly explained by the fact that PrEP initiation and continuation relied solely on public sector ART trained nurses and counsellors, who may be overstretched with their work with women living with HIV. Furthermore, PrEP continuation may have been underestimated as it only looks at continuation for women going back to the same clinic. If the woman went to a different clinic for her PrEP refill, or if she had to continue her postpartum care in a different clinic than the delivery clinic, it would not be captured in our data. In South Africa, pregnant women are seen at least four times whilst pregnant, and high proportions of PrEP continuation are likely linked to frequent antenatal visits [42]. For breastfeeding women, because of the lack of integration of maternal and child services, their point of care was not well-defined. Potential solutions include mothers seen as a dyad with their infant, use of HIV self-testing services, or alternatively providing PrEP within family planning and sexual and reproductive care settings.

There were very few primary care clinics in the surrounding areas providing PrEP for PBFW (other than the eight we trained), which meant that once women had delivered their babies, they often struggled to find a clinic to continue PrEP. Finally, within a same clinic, PrEP was not readily offered in other services such as family planning. These challenges emphasise the need for future research focusing on understanding unique health system barriers and facilitators to PrEP initiation and continuation among PBFW, especially for those postpartum.

There was good buy-in and support from participating clinics and adequate support from the local health authorities. The proportion of trained providers who counselled and provided PrEP according to the training and to the DOH guidelines was good. Although mentorship was only provided to nurses and counsellors actively counselling and providing PrEP to PBFW, we trained a much larger portion of clinic staff, with the aim of increasing PrEP knowledge in the community and in cadres who are frontline workers, providing informal health promotion. Furthermore, by highlighting the evidence on PrEP safety in PBFW and increasing PrEP awareness among HCPs, we hoped to increase their confidence and ability to better implement PrEP, as found by other colleagues [43]. We found that the staff was eager to learn about PrEP. Although the pre-training tests results were often quite low, confirming that HCP PrEP knowledge was poor prior to the training, the post-training questionnaires increased by a median of 31% [19]. Whilst assessment checklist scores varied over time by provider cadre, scores overall were predominately static from start to end of mentorship. Our findings reinforce that of other studies, showing that training HCP on PrEP improved HIV counselling, PrEP screening, uptake, and adherence [22, 36, 43].

Although we only included the eight clinics where we were providing the implementation science study, we found that, at the time of our study, the implementation of PrEP for PBFW had not started in other clinics in the same subdistrict in Cape Town [29]. Effective implementation of new guidelines often comes with new monitoring tools, such as national indicators [24]. In April 2023, the DOH announced new national indicators, including indicators tracking PrEP initiation and continuation in ANC. The implementation of these new indicators may have contributed to the increases observed in PrEP initiation between June and September 2023. However, there is still no indicator for postpartum or breastfeeding women which may continue to negatively impact both PrEP initiation and continuation after childbirth.

All study clinics were still delivering PrEP to PBFW 3 months after the intervention. The overall mean monthly number of PrEP initiations in pregnancy whilst mentoring was 85 and declined to 78 without our regular study mentoring visits. A decline in number of initiations once mentoring support ended is to be expected [22]. It was encouraging to see that the overall number of monthly PrEP initiations in breastfeeding women decreased by only 4 (21%) after handover. Many factors could have played a role in this improvement, including community PrEP knowledge increasing during the time of our implementation, resulting in an increase in PrEP demand and uptake [44–46]. Secondly, during the timing of our implementation, we worked in partnership with the local health authorities and highlighted some of the difficulties we encountered on the ground. This led to workshops with the clinics, with the support of the local authorities, and led to some significant changes in the clinic flow, particularly for breastfeeding women. For example, one clinic decided to test all mothers at the 14-week immunisation visit to facilitate offering of PrEP, on top of the normal 6-week testing. Another clinic implemented an appointment system for the mother and babies at 3-monthly interval to improve maternal HIV testing. These changes could have resulted in improvement in HIV testing and PrEP offer to breastfeeding women and outbalanced the decrease in PrEP initiation stemming from the withdrawal of mentorship and support.

The key strength is the real-world implementation of the study with limited study involvement or additional PrEP dedicated staff aside from a small training and mentoring study team working in collaboration with DOH staff. We used real-world programme data which is crucial to understand implementation. This increased the generalisability of our study findings to other similar settings. Limitations include reduced capacity for collection of additional data beyond routine aggregated data collected by DOH staff. For example, although we could identify how many pregnant women were tested for HIV, it was challenging to ascertain how many of them were offered PrEP. It was also challenging to ascertain how many breastfeeding women accompanied their babies to their immunisation visits and were tested for HIV as no indicators are collected. Finally, our maintenance ascertainment was limited to 3-month follow-up and may be insufficient to assess longer-term maintenance. These findings highlight the need for revised documentation and data capturing systems within the clinics and DOH, which include PrEP indicators that are inclusive of those postpartum, to ensure better concordance with PrEP guidelines and improved PrEP implementation.

## Conclusions

In Cape Town, South Africa, the proportion of PBFW initiating and continuing PrEP rapidly increased but was lower than expected when compared to other research studies. Nursing staff and counsellors benefited from supportive training to follow PrEP integration. Barriers to the integration of PrEP, particularly for breastfeeding women, were observed and included limited, opt-in repeat HIV testing of breastfeeding mothers and need for additional ART-trained nurses to prescribe PrEP. Enablers included motivated and dedicated staff. Our study contributes to literature that demonstrates the importance of training and staff support to improve the integration of PrEP into antenatal and postnatal care settings. More research is needed to understand how best to improve PrEP initiation and continuation in not only pregnant but also postpartum and breastfeeding women, at risk of HIV.

## Supplementary Information

Additional file 1. Mentoring assessments for nurses and for counsellors

Additional file 2. Codebook

Additional file 3. Dataset

Additional file 4. Adoption of PrEP guidelines by healthcare providers measured by post-test questionnaire results and mentoring score

Additional file 5. Demographics of healthcare providers who completing the PrEP training and mentorship (April 2022- January 2023)

## Data Availability

The data that support the findings of this study are available from the study as supplementary materials including the study data and codebook (Additional Files 2 and 3). For additional information, please contact the study PI, Dvora Joseph Davey (dvora.josephdavey@uct.ac.za).  Data is provided within the manuscript or supplementary information files.

## Abbreviations

ANC: Antenatal care
ART: Antiretroviral therapy
CHC: Community health centre
CCT: City of Cape Town
CI: Confidence interval
CNP: Clinical nurse practitioner
DOH: Department of Health
HCP: Healthcare provider
HIV: Human immunodeficiency virus
MCH: Maternal and child health
MHS: Metro Health Services
MOU: Midwifery obstetric unit
NGO: Non-governmental organizations
NIMART: Nurse-initiation and management of ART
PN: Professional nurse
PNC: Postnatal care
PrEP: Pre-exposure prophylaxis
RE-AIM: Reach, Effectiveness, Adoption, Implementation, and Maintenance
UCT: University of Cape Town
VT: Vertical transmission
WHO: World Health Organization
WLHIV: Women living with HIV
