# Supplementary figures and images for: Integration of HIV pre-exposure prophylaxis (PrEP) services for pregnant and breastfeeding women in eight primary care clinics: results of an implementation science study
Source: BMC Glob Public Health. 2024 Aug 26;2:57. doi: 10.1186/s44263-024-00089-8 (PMC11622949; doi:10.1186/s44263-024-00089-8)

**Figure S1. Mentoring assessments for nurses and for counsellors**


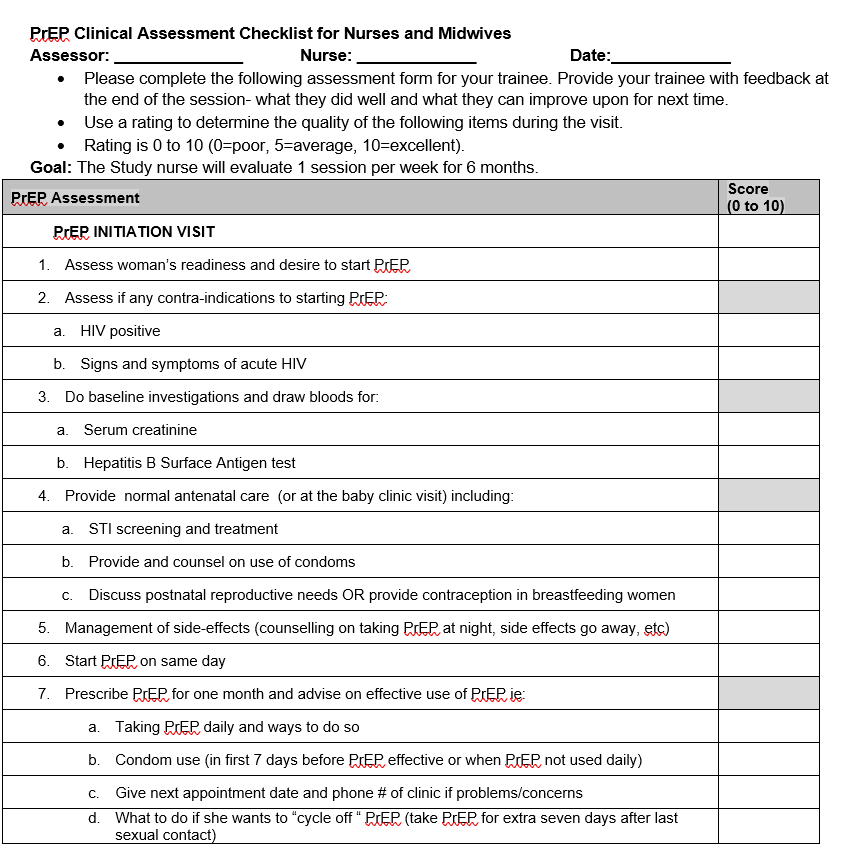


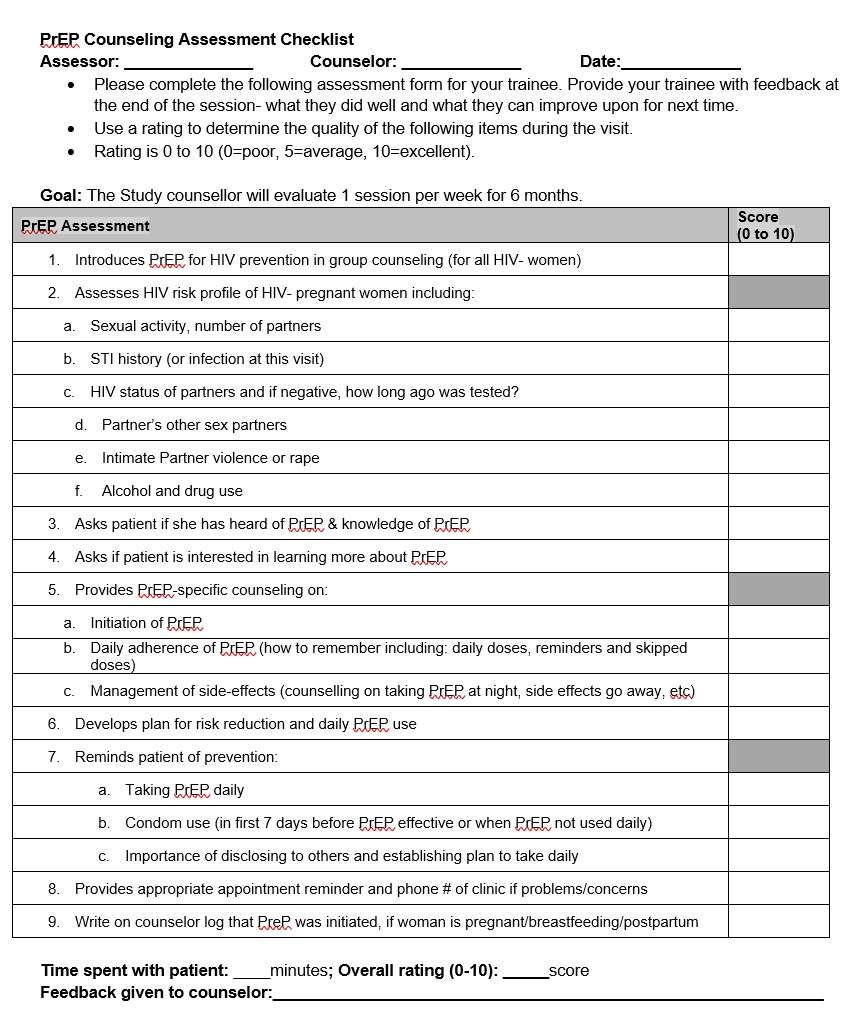

Supplement: Supplementary file 1 — Additional file 1. Mentoring assessments for nurses and for counsellors [file 44263_2024_89_MOESM1_ESM.docx]
