# Supplementary material for: Integration of HIV pre-exposure prophylaxis (PrEP) services for pregnant and breastfeeding women in eight primary care clinics: results of an implementation science study
Source: BMC Glob Public Health. 2024 Aug 26;2:57. doi: 10.1186/s44263-024-00089-8 (PMC11622949; doi:10.1186/s44263-024-00089-8)
Supplement: Supplementary file 2 — Additional file 2. Codebook [file 44263_2024_89_MOESM2_ESM.pdf]

# Data Dictionary Codebook

09.05.2024 14:35

| # | Variable / Field Name | Field Label *Field Note* | Field Attributes (Field Type, Validation, Choices, Calculations, etc.) |
|---|---|---|---|
| **Instrument: PID (pid)** | | | |
| 1 | [pid] | Record ID | text |
| 2 | [pid_complete] | Section Header: *Form Status*<br>Complete? | dropdown<br>0 Incomplete<br>1 Unverified<br>2 Complete |
| **Instrument: Clinic Information (clinic_information)** | | | |
| 3 | [clinic_name] | Clinic name | dropdown, Required<br>1 Gugulethu MOU<br>2 Gugulethu Clinic<br>3 Gugulethu CHC<br>4 Hanover Park MOU<br>5 Hanover Park Clinic<br>6 Mitchells Plain MOU<br>7 Mitchells Plain Clinic<br>8 Vuyani MOU<br>9 Vuyani Clinic<br>10 Nyanga MOU<br>11 Nyanga CHC<br>12 Crossroads MOU<br>13 Crossroads CHC<br>14 Inzame Zabantu MOU<br>15 Inzame Zabantu CHC<br>16 Other |
| 4 | [clinic_name_1]<br>Show the field ONLY if:<br>[clinic_name] = '16' | If other, specify: | text |
| 5 | [clinic_org] | Organisation name | dropdown, Required<br>1 Anova<br>2 CCT (includes Men clinic)<br>3 TBHIV |

| | | | |
|---|---|---|---|
| | | | 4 DOH (Dept. of Health)<br>5 Other |
| 6 | [clinic_org_1]<br><br>Show the field ONLY if:<br>[clinic_org] = '5' | If other, specify: | text, Required |
| 7 | [clinic_role] | Role | dropdown, Required<br>1 Professional Nurse<br>2 Certified Nurse Practitioner<br>3 Nurse clinician<br>12 Enrolled nurse<br>13 Enrolled Nurse Assistant (ENA)<br>4 Counsellor (includes HAST counsellor)<br>5 Health promoter<br>6 Midwife<br>7 Advanced midwife<br>8 Pharmacist<br>9 Pharmacy assistant<br>10 Clinic Manager<br>11 Other |
| 8 | [clinic_role_1]<br><br>Show the field ONLY if:<br>[clinic_role] = '11' | If other, specify: | text, Required |
| 9 | [clinic_comment] | Any other comments | notes |
| 10 | [clinic_initials] | Capturer's initials | text (alpha_only), Required |
| 11 | [clinic_information_complete] | Section Header: *Form Status*<br>Complete? | dropdown<br>0 Incomplete<br>1 Unverified<br>2 Complete |
| **Instrument: Demographics Questionnaire (demographics_questionnaire)** | | | |
| 12 | [demo_date] | Date | text (date_dmy, Min: 2022-03-01), Required |
| 13 | [demo_q1] | 1. For how many years have you been working in this role (e.g. midwife, counselor, health promoter, nurse, manager etc)?<br>*Years* | text (number, Min: 0), Required |
| 14 | [demo_q2] | 2. What are your highest qualifications? | dropdown, Required<br>1 Bachelor of Nursing<br>2 Bachelor of Technology |

| | | | |
|---|---|---|---|
| | | | 3 Diploma<br>4 Matric<br>5 Other |
| 15 | [demo_q2_1]<br>Show the field ONLY if:<br>[demo_q2] = '5' | If other, specify: | text, Required |
| 16 | [demo_q3] | 3. Have you done the NDOH PreP online training? | yesno, Required<br>1 Yes<br>0 No |
| 17 | [demo_q4] | 4. Have you ever worked with PrEP before? | yesno, Required<br>1 Yes<br>0 No |
| 18 | [demo_q4_1]<br>Show the field ONLY if:<br>[demo_q4] = '1' | 4.1. If yes, for how long (years)?<br>*Years* | text (number, Min: 0) |
| 19 | [demo_q5] | 5. If you are a nurse, are you? | dropdown, Required<br>1 EN<br>2 PN<br>3 CNP<br>4 Midwife<br>5 Advanced midwife<br>7 Other<br>6 Not a nurse |
| 20 | [demo_q5_1]<br>Show the field ONLY if:<br>[demo_q5] = '7' | 5.1. If other, specify:<br>*Years* | text |
| 21 | [demo_q6] | 6. If you are a nurse, are you NIMARTed ? | radio, Required<br>1 Yes<br>0 No<br>98 N/A (not a nurse) |
| 22 | [demo_q7] | 7. What is your gender? | radio, Required<br>0 Male<br>1 Female<br>99 Prefer not to answer |
| 23 | [demo_comment] | Any other comments | notes |
| 24 | [demo_initials] | Capturer's initials | text, Required |

| | | | |
|---|---|---|---|
| 25 | [demographics_questionnaire_complete] | Section Header: *Form Status*<br>Complete? | dropdown<br>0 Incomplete<br>1 Unverified<br>2 Complete |
| **Instrument: Pre-Training Test (pretraining_test)** | | | |
| 26 | [pre_test_date] | Date | text (date_dmy, Min: 2022-03-01), Required |
| 27 | [pre_test_q1] | 1. Is the following statement true or false?<br><br>"PrEP is safe to use during pregnancy and breastfeeding."<br><br>Circle the correct answer | radio, Required<br>1 a. True<br>0 b. False |
| 28 | [pre_test_q1_score] | Answer: A | radio, Required<br>1 Correct<br>0 Incorrect |
| 29 | [pre_test_q2] | 2. Why is it important to offer PrEP to pregnant and breastfeeding women?<br><br>Circle the correct answer | radio, Required<br>1 a. Women are at increased risk of HIV acquisition during pregnancy and breastfeeding<br>2 b. PrEP is more effective in pregnant women than it is in women who are not pregnant.<br>3 c. Women are less likely to agree to take PrEP during pregnancy than they are at other times. |
| 30 | [pre_test_q2_score] | Answer: A | radio, Required<br>1 Correct<br>0 Incorrect |
| 31 | [pre_test_q3] | 3. Which common medications prescribed during pregnancy can have unwanted interactions with PrEP?<br><br>Circle the correct answer | radio, Required<br>1 a. PrEP may interact with antenatal medications such as iron and folic acid tablets.<br>2 b. PrEP may interact with malaria treatments, including sulfadoxine-pyrimethamine.<br>3 c. The medications used in PrEP have no known drug interactions with the most commonly prescribed pregnancy medications. |
| 32 | [pre_test_q3_score] | Answer: C | radio, Required<br>1 Correct<br>0 Incorrect |

| | | | |
|---|---|---|---|
| 33 | [pre_test_q4] | 4. Which of the following are contraindications to starting PrEP in pregnant women?<br><br>Select all that apply. | checkbox, Required<br>1 pre_test_q4___1 a. Creatinine of more than 85 umol/l<br>2 pre_test_q4___2 b. HIV infection or signs/symptoms of acute HIV infection<br>3 pre_test_q4___3 c. PrEP use prior to pregnancy<br>4 pre_test_q4___4 d. Hepatitis B infection |
| 34 | [pre_test_q4_score] | Answer: A & B | radio, Required<br>1 Correct<br>0 Incorrect |
| 35 | [pre_test_q5] | 5. Which of the following is a recommended approach to monitoring kidney function for PrEP users who are pregnant?<br><br>Circle the correct answer | radio, Required<br>1 a. Monitor serum creatinine or creatinine clearance every two weeks.<br>2 b. Monitor serum creatinine or creatinine clearance every month.<br>3 c. Monitor serum creatinine or creatinine clearance at baseline, after 3 months and 7 months |
| 36 | [pre_test_q5_score] | Answer: C | radio, Required<br>1 Correct<br>0 Incorrect |
| 37 | [pre_test_q6] | 6. Is the following statement true or false?<br><br>"PrEP and post-exposure prophylaxis (PEP) are both used by HIV-negative persons to prevent HIV acquisition"<br><br>Circle the correct answer | radio, Required<br>1 a. True<br>0 b. False |
| 38 | [pre_test_q6_score] | Answer: A | radio, Required<br>1 Correct<br>0 Incorrect |
| 39 | [pre_test_q7] | 7. Is the following statement true or false?<br><br>"PrEP is protective against a variety of sexually transmitted infections."<br><br>Circle the correct answer | radio, Required<br>1 a. True<br>0 b. False |

| | | | |
|---|---|---|---|
| 40 | [pre_test_q7_score] | Answer: B | radio, Required<br>1 Correct<br>0 Incorrect |
| 41 | [pre_test_q8] | 8. What should the provider do if the client stated PrEP was not used continually and reported imperfect PrEP use?<br><br>Circle the correct answer | radio, Required<br>1 a. Daily PrEP use is not important<br>2 b. Remind client to use condoms until PrEP has been taken for 7 days<br>3 c. Remind client to use condoms until PrEP has been taken for 28 days |
| 42 | [pre_test_q8_score] | Answer: B | radio, Required<br>1 Correct<br>0 Incorrect |
| 43 | [pre_test_q9] | 9. Which of the following is false when counseling pregnant and breastfeeding women?<br><br>Circle the false answer | radio, Required<br>1 a. PrEP use during pregnancy does not cause babies to be too big or too small.<br>2 b. PrEP may affect a woman's future fertility<br>3 c. PrEP does not affect a mother's milk production or the taste or quality of breast milk. |
| 44 | [pre_test_q9_score] | Answer: B | radio, Required<br>1 Correct<br>0 Incorrect |
| 45 | [pre_test_q10] | 10. Which of the following statements are true?<br><br>Select all that apply | checkbox, Required<br>1 pre_test_q10___1 a. PrEP side effects include fainting, weight loss and loss of smell<br>2 pre_test_q10___2 b. PrEP side effects ago away within a few weeks for most people<br>3 pre_test_q10___3 c. 90% of PrEP users will have no side effects<br>4 pre_test_q10___4 d. PrEP side effects are similar to |

| | | | |
|---|---|---|---|
| | | | symptoms of pregnancy |
| 46 | [pre_test_q10_score] | Answer: B, C & D | radio, Required<br>1 Correct<br>0 Incorrect |
| 47 | [pre_test_score] | Total score | calc, Required<br>Calculation: sum([pre_test_q1_score], [pre_test_q2_score], [pre_test_q3_score], [pre_test_q4_score], [pre_test_q5_score], [pre_test_q6_score], [pre_test_q7_score], [pre_test_q8_score], [pre_test_q9_score], [pre_test_q10_score]) |
| 48 | [pre_test_result] | Result (Pass: 8/10) | radio, Required<br>1 Pass<br>0 Fail |
| 49 | [pre_test_comment] | Any other comments | notes |
| 50 | [pre_test_initials] | Capturer's initials | text, Required |
| 51 | [pretraining_test_complete] | Section Header: *Form Status*<br>Complete? | dropdown<br>0 Incomplete<br>1 Unverified<br>2 Complete |
| **Instrument: Post-Training Test (posttraining_test)** | | | |
| 52 | [post_test_date] | Date | text (date_dmy, Min: 2022-03-01), Required |
| 53 | [post_test_q1] | 1. Is the following statement true or false?<br><br>"PrEP is safe to use during pregnancy and breastfeeding."<br><br>Circle the correct answer | radio, Required<br>1 a. True<br>0 b. False |
| 54 | [post_test_q1_score] | Answer: A | radio, Required<br>1 Correct<br>0 Incorrect |
| 55 | [post_test_q2] | 2. Why is it important to offer PrEP to pregnant and breastfeeding women?<br><br>Circle the correct answer | radio, Required<br>1 a. Women are at increased risk of HIV acquisition during pregnancy and breastfeeding<br>2 b. PrEP is more effective in pregnant women than it is in women who are not pregnant. |

| | | | |
|---|---|---|---|
| | | | 3 c. Women are less likely to agree to take PrEP during pregnancy than they are at other times. |
| 56 | [post_test_q2_score] | Answer: A | radio, Required<br>1 Correct<br>0 Incorrect |
| 57 | [post_test_q3] | 3. Which common medications prescribed during pregnancy can have unwanted interactions with PrEP?<br><br>Circle the correct answer | radio, Required<br>1 a. PrEP may interact with antenatal medications such as iron and folic acid tablets.<br>2 b. PrEP may interact with malaria treatments, including sulfadoxine-pyrimethamine.<br>3 c. The medications used in PrEP have no known drug interactions with the most commonly prescribed pregnancy medications. |
| 58 | [post_test_q3_score] | Answer: C | radio, Required<br>1 Correct<br>0 Incorrect |
| 59 | [post_test_q4] | 4. Which of the following are contraindications to starting PrEP in pregnant women?<br><br>Select all that apply. | checkbox, Required<br>1 post_test_q4___1 a. Creatinine of more than 85 umol/l<br>2 post_test_q4___2 b. HIV infection or signs/symptoms of acute HIV infection<br>3 post_test_q4___3 c. PrEP use prior to pregnancy<br>4 post_test_q4___4 d. Hepatitis B infection |
| 60 | [post_test_q4_score] | Answer: A & B | radio, Required<br>1 Correct<br>0 Incorrect |
| 61 | [post_test_q5] | 5. Which of the following is a recommended approach to monitoring kidney function for PrEP users who are pregnant?<br><br>Circle the correct answer | radio, Required<br>1 a. Monitor serum creatinine or creatinine clearance every two weeks.<br>2 b. Monitor serum creatinine or creatinine clearance every month. |

| | | | |
|---|---|---|---|
| | | | 3 c. Monitor serum creatinine or creatinine clearance at baseline, after 3 months and 7 months |
| 62 | [post_test_q5_score] | Answer: C | radio, Required<br>1 Correct<br>0 Incorrect |
| 63 | [post_test_q6] | 6. Is the following statement true or false?<br><br>"PrEP and post-exposure prophylaxis (PEP) are both used by HIV-negative persons to prevent HIV acquisition"<br><br>Circle the correct answer | radio, Required<br>1 a. True<br>0 b. False |
| 64 | [post_test_q6_score] | Answer: A | radio, Required<br>1 Correct<br>0 Incorrect |
| 65 | [post_test_q7] | 7. Is the following statement true or false?<br><br>"PrEP is protective against a variety of sexually transmitted infections."<br><br>Circle the correct answer | radio, Required<br>1 a. True<br>0 b. False |
| 66 | [post_test_q7_score] | Answer: B | radio, Required<br>1 Correct<br>0 Incorrect |
| 67 | [post_test_q8] | 8. What should the provider do if the client stated PrEP was not used continually and reported imperfect PrEP use?<br><br>Circle the correct answer | radio, Required<br>1 a. Daily PrEP use is not important<br>2 b. Remind client to use condoms until PrEP has been taken for 7 days<br>3 c. Remind client to use condoms until PrEP has been taken for 28 days |
| 68 | [post_test_q8_score] | Answer: B | radio, Required<br>1 Correct<br>0 Incorrect |
| 69 | [post_test_q9] | 9. Which of the following is false when counseling pregnant and breastfeeding women?<br><br>Circle the false answer | radio, Required<br>1 a. PrEP use during pregnancy does not cause babies to be too big or too small.<br>2 b. PrEP may affect a woman's future fertility<br>3 c. PrEP does not affect a mother's milk production or the taste or quality of breast milk. |

| | | | |
|---|---|---|---|
| 70 | [post_test_q9_score] | Answer: B | radio, Required<br>1 Correct<br>0 Incorrect |
| 71 | [post_test_q10] | 10. Which of the following statements are true?<br><br>Select all that apply | checkbox, Required<br>1 post_test_q10___1 a. PrEP side effects include fainting, weight loss and loss of smell<br>2 post_test_q10___2 b. PrEP side effects ago away within a few weeks for most people<br>3 post_test_q10___3 c. 90% of PrEP users will have no side effects<br>4 post_test_q10___4 d. PrEP side effects are similar to symptoms of pregnancy |
| 72 | [post_test_q10_score] | Answer: B, C & D | radio, Required<br>1 Correct<br>0 Incorrect |
| 73 | [post_test_score] | Total score | calc, Required<br>Calculation: sum([post_test_q1_score], [post_test_q2_score], [post_test_q3_score], [post_test_q4_score], [post_test_q5_score], [post_test_q6_score], [post_test_q7_score], [post_test_q8_score], [post_test_q9_score], [post_test_q10_score]) |
| 74 | [post_test_result] | Result (Pass: 8/10) | radio, Required<br>1 Pass<br>0 Fail |
| 75 | [post_test_review]<br><br>Show the field ONLY if: [post_test_result] = '0' | Section Header: *FOR UCT STAFF:*<br><br>FOR UCT STAFF: If failed on the questionnaire (less than 80%), was the questionnaire reviewed with the counsellor/nurse? | yesno<br>1 Yes<br>0 No |

| | | | |
|---|---|---|---|
| 76 | [post_test_comment] | Any other comments | notes |
| 77 | [post_test_initials] | Capturer's initials | text, Required |
| 78 | [posttraining_test_complete] | Section Header: *Form Status*<br>Complete? | dropdown<br>0 Incomplete<br>1 Unverified<br>2 Complete |
| **Instrument: PrEP Counsellor Assessment Checklist (prep_counsellor_assessment_checklist)** | | | |
| 79 | [counsellor_date] | Date | text (date_dmy, Min: 2022-03-01), Required |
| 80 | [counsellor_assessor] | Assessor | text, Required |
| 81 | [counsellor_q1] | 1. Introduces PrEP for HIV prevention in group counseling (for all HIV- women) | dropdown, Required<br>0 0<br>1 1<br>2 2<br>3 3<br>4 4<br>5 5<br>6 6<br>7 7<br>8 8<br>9 9<br>10 10 |
| 82 | [counsellor_q2a] | Section Header: *Assesses HIV risk profile of HIV-pregnant women including:*<br>2a. Sexual activity, number of partners | dropdown, Required<br>0 0<br>1 1<br>2 2<br>3 3<br>4 4<br>5 5<br>6 6<br>7 7<br>8 8<br>9 9<br>10 10 |
| 83 | [counsellor_q2b] | 2b. STI history (or infection at this visit) | dropdown, Required<br>0 0<br>1 1 |

| | | | |
|---|---|---|---|
| | | | 2 2<br>3 3<br>4 4<br>5 5<br>6 6<br>7 7<br>8 8<br>9 9<br>10 10 |
| 84 | [ counsellor_q2c ] | 2c. HIV status of partners and if negative, how long ago was tested? | dropdown, Required<br>0 0<br>1 1<br>2 2<br>3 3<br>4 4<br>5 5<br>6 6<br>7 7<br>8 8<br>9 9<br>10 10 |
| 85 | [ counsellor_q2d ] | 2d. Partner's other sex partners | dropdown, Required<br>0 0<br>1 1<br>2 2<br>3 3<br>4 4<br>5 5<br>6 6<br>7 7<br>8 8<br>9 9<br>10 10 |
| 86 | [ counsellor_q2e ] | 2e. Intimate Partner violence or rape | dropdown, Required<br>0 0<br>1 1<br>2 2<br>3 3 |

| | | | |
|---|---|---|---|
| | | | 4 4<br>5 5<br>6 6<br>7 7<br>8 8<br>9 9<br>10 10 |
| 87 | [counsellor_q2f] | 2f. Alcohol and drug use | dropdown, Required<br>0 0<br>1 1<br>2 2<br>3 3<br>4 4<br>5 5<br>6 6<br>7 7<br>8 8<br>9 9<br>10 10 |
| 88 | [counsellor_q3] | 3. Asks patient if she has heard of PrEP & knowledge of PrEP | dropdown, Required<br>0 0<br>1 1<br>2 2<br>3 3<br>4 4<br>5 5<br>6 6<br>7 7<br>8 8<br>9 9<br>10 10 |
| 89 | [counsellor_q4] | 4. Asks if patient is interested in learning more about PrEP | dropdown, Required<br>0 0<br>1 1<br>2 2<br>3 3<br>4 4<br>5 5 |

| | | | |
|---|---|---|---|
| | | | 6 6<br>7 7<br>8 8<br>9 9<br>10 10 |
| 90 | [counsellor_q5a] | Section Header: *Provides PrEP-specific counseling on:*<br>5a. Initiation of PrEP | dropdown, Required<br>0 0<br>1 1<br>2 2<br>3 3<br>4 4<br>5 5<br>6 6<br>7 7<br>8 8<br>9 9<br>10 10 |
| 91 | [counsellor_q5b] | 5b. Daily adherence of PrEP (how to remember including: daily doses, reminders and skipped doses) | dropdown, Required<br>0 0<br>1 1<br>2 2<br>3 3<br>4 4<br>5 5<br>6 6<br>7 7<br>8 8<br>9 9<br>10 10 |
| 92 | [counsellor_q5c] | 5c. Management of side-effects (counselling on taking PrEP at night, side effects go away, etc) | dropdown, Required<br>0 0<br>1 1<br>2 2<br>3 3<br>4 4<br>5 5<br>6 6<br>7 7 |

| | | | |
|---|---|---|---|
| | | | 8 8<br>9 9<br>10 10 |
| 93 | [counsellor_q6] | 6. Develops plan for risk reduction and daily PrEP use | dropdown, Required<br>0 0<br>1 1<br>2 2<br>3 3<br>4 4<br>5 5<br>6 6<br>7 7<br>8 8<br>9 9<br>10 10 |
| 94 | [counsellor_q7a] | Section Header: *Reminds patient of prevention:*<br>7a. Taking PrEP daily | dropdown, Required<br>0 0<br>1 1<br>2 2<br>3 3<br>4 4<br>5 5<br>6 6<br>7 7<br>8 8<br>9 9<br>10 10 |
| 95 | [counsellor_q7b] | 7b. Condom use (in first 7 days before PrEP effective or when PrEP not used daily) | dropdown, Required<br>0 0<br>1 1<br>2 2<br>3 3<br>4 4<br>5 5<br>6 6<br>7 7<br>8 8<br>9 9 |

| | | | |
|---|---|---|---|
| | | | 10 10 |
| 96 | [counsellor_q7c] | 7c. Importance of disclosing to others and establishing plan to take daily | dropdown, Required<br>0 0<br>1 1<br>2 2<br>3 3<br>4 4<br>5 5<br>6 6<br>7 7<br>8 8<br>9 9<br>10 10 |
| 97 | [counsellor_q8] | 8. Provides appropriate appointment reminder and phone # of clinic if problems/concerns | dropdown, Required<br>0 0<br>1 1<br>2 2<br>3 3<br>4 4<br>5 5<br>6 6<br>7 7<br>8 8<br>9 9<br>10 10 |
| 98 | [counsellor_q9] | 9. Write on counselor log that PrEP was initiated if woman is pregnant/breastfeeding/postpartum | dropdown, Required<br>0 0<br>1 1<br>2 2<br>3 3<br>4 4<br>5 5<br>6 6<br>7 7<br>8 8<br>9 9<br>10 10 |
| 99 | [counsellor_time] | Time spent with patient (minutes): | text (number, Min: 0), Required |

| | | | |
|---|---|---|---|
| | | *Minutes* | |
| 100 | [counsellor_overall] | Overall rating (0-10): | dropdown, Required<br>0 0<br>1 1<br>2 2<br>3 3<br>4 4<br>5 5<br>6 6<br>7 7<br>8 8<br>9 9<br>10 10 |
| 101 | [counsellor_feedback] | Feedback given to counselor: | notes, Required |
| 102 | [counsellor_comment] | Any other comments | notes |
| 103 | [counsellor_initials] | Capturer's initials | text, Required |
| 104 | [prep_counsellor_assessment_checklist_complete] | Section Header: *Form Status*<br>Complete? | dropdown<br>0 Incomplete<br>1 Unverified<br>2 Complete |

**Instrument: PrEP Initiation Nurse Assessment Checklist (prep_initiation_nurse_assessment_checklist)**

| | | | |
|---|---|---|---|
| 105 | [n_initiate_date] | Date | text (date_dmy, Min: 2022-03-01), Required |
| 106 | [n_initiate_assessor] | Assessor | text, Required |
| 107 | [n_initiate_q1] | 1. Assess woman's readiness and desire to start PrEP | dropdown, Required<br>0 0<br>1 1<br>2 2<br>3 3<br>4 4<br>5 5<br>6 6<br>7 7<br>8 8<br>9 9 |

| | | | |
|---|---|---|---|
| | | | 10 10 |
| 108 | [n_initiate_q2a] | Section Header: *Assess if any contra-indications to starting PrEP:*<br>2a. HIV positive | dropdown, Required<br>0 0<br>1 1<br>2 2<br>3 3<br>4 4<br>5 5<br>6 6<br>7 7<br>8 8<br>9 9<br>10 10 |
| 109 | [n_initiate_q2b] | 2b. Signs and symptoms of acute HIV | dropdown, Required<br>0 0<br>1 1<br>2 2<br>3 3<br>4 4<br>5 5<br>6 6<br>7 7<br>8 8<br>9 9<br>10 10 |
| 110 | [n_initiate_q3a] | Section Header: *Do baseline investigations and draw bloods for:*<br>3a. Serum creatinine | dropdown, Required<br>0 0<br>1 1<br>2 2<br>3 3<br>4 4<br>5 5<br>6 6<br>7 7<br>8 8<br>9 9<br>10 10 |
| 111 | [n_initiate_q3b] | 3b. Hepatitis B Surface Antigen test | dropdown, Required |

| | | | |
|---|---|---|---|
| | | | 0 0<br>1 1<br>2 2<br>3 3<br>4 4<br>5 5<br>6 6<br>7 7<br>8 8<br>9 9<br>10 10 |
| 112 | [n_initiate_q4a] | Section Header: *Provide normal antenatal care (or at the baby clinic visit) including:*<br>4a. STI screening and treatment | dropdown, Required<br>0 0<br>1 1<br>2 2<br>3 3<br>4 4<br>5 5<br>6 6<br>7 7<br>8 8<br>9 9<br>10 10 |
| 113 | [n_initiate_q4b] | 4b. Provide and counsel on use of condoms | dropdown, Required<br>0 0<br>1 1<br>2 2<br>3 3<br>4 4<br>5 5<br>6 6<br>7 7<br>8 8<br>9 9<br>10 10 |
| 114 | [n_initiate_q4c] | 4c. Discuss postnatal reproductive needs OR provide contraception in breastfeeding women | dropdown, Required<br>0 0<br>1 1 |

| | | | |
|---|---|---|---|
| | | | 2 2<br>3 3<br>4 4<br>5 5<br>6 6<br>7 7<br>8 8<br>9 9<br>10 10 |
| 115 | [n_initiate_q5] | 5. Management of side-effects (counselling on taking PrEP at night, side effects go away, etc) | dropdown, Required<br>0 0<br>1 1<br>2 2<br>3 3<br>4 4<br>5 5<br>6 6<br>7 7<br>8 8<br>9 9<br>10 10 |
| 116 | [n_initiate_q6] | 6. Start PrEP on same day | dropdown, Required<br>0 0<br>1 1<br>2 2<br>3 3<br>4 4<br>5 5<br>6 6<br>7 7<br>8 8<br>9 9<br>10 10 |
| 117 | [n_initiate_q7a] | Section Header: *Prescribe PrEP for one month and advise on effective use of PrEP ie:*<br>7a. Taking PrEP daily and ways to do so | dropdown, Required<br>0 0<br>1 1<br>2 2<br>3 3 |

| | | | |
|---|---|---|---|
| | | | 4 4<br>5 5<br>6 6<br>7 7<br>8 8<br>9 9<br>10 10 |
| 118 | [n_initiate_q7b] | 7b. Condom use (in first 7 days before PrEP effective or when PrEP not used daily) | dropdown, Required<br>0 0<br>1 1<br>2 2<br>3 3<br>4 4<br>5 5<br>6 6<br>7 7<br>8 8<br>9 9<br>10 10 |
| 119 | [n_initiate_q7c] | 7c. What to do if she wants to "cycle of PrEP (take PrEP for extra seven days after last sexual contact) | dropdown, Required<br>0 0<br>1 1<br>2 2<br>3 3<br>4 4<br>5 5<br>6 6<br>7 7<br>8 8<br>9 9<br>10 10 |
| 120 | [n_initiate_q7d] | 7d. Ensure prescription is signed/counter signed by NIMART nurse | dropdown, Required<br>0 0<br>1 1<br>2 2<br>3 3<br>4 4<br>5 5 |

| | | | |
|---|---|---|---|
| | | | 6 6<br>7 7<br>8 8<br>9 9<br>10 10 |
| 121 | [n_initiate_q8] | 8. Give next appointment date and phone # of clinic if problems/concerns | dropdown<br>1 1<br>2 2<br>3 3<br>4 4<br>5 5<br>6 6<br>7 7<br>8 8<br>9 9<br>10 10 |
| 122 | [n_initiate_q9] | 9. Enter all relevant information in the stationary (PrEP on CMR, use of integrated stationery) | dropdown<br>1 1<br>2 2<br>3 3<br>4 4<br>5 5<br>6 6<br>7 7<br>8 8<br>9 9<br>10 10 |
| 123 | [n_initiate_time] | Time spent with patient (minutes):<br>*Minutes* | text (number, Min: 0), Required |
| 124 | [n_initiate_overall] | Overall rating (0-10): | dropdown, Required<br>0 0<br>1 1<br>2 2<br>3 3<br>4 4<br>5 5<br>6 6<br>7 7<br>8 8 |

| | | | |
|---|---|---|---|
| | | | 9 9<br>10 10 |
| 125 | [n_initiate_feedback] | Feedback given to nurse: | notes, Required |
| 126 | [n_initiate_comment] | Any other comments | notes |
| 127 | [n_initiate_initials] | Capturer's initials | text, Required |
| 128 | [prep_initiation_nurse_assessment_checklist_complete] | Section Header: *Form Status*<br>Complete? | dropdown<br>0 Incomplete<br>1 Unverified<br>2 Complete |

**Instrument: PrEP Follow-up Nurse Assessment Checklist (prep_followup_nurse_assessment_checklist)**

| | | | |
|---|---|---|---|
| 129 | [n_followup_date] | Date | text (date_dmy, Min: 2022-03-01), Required |
| 130 | [n_followup_assessor] | Assessor | text, Required |
| 131 | [n_followup_q1a] | Section Header: *Review HIV test result:*<br>1a. If negative, continue PrEP | dropdown, Required<br>0 0<br>1 1<br>2 2<br>3 3<br>4 4<br>5 5<br>6 6<br>7 7<br>8 8<br>9 9<br>10 10 |
| 132 | [n_followup_q1b] | 1b. If positive, stop PrEP immediately and manage for ART initiation on same day | dropdown, Required<br>0 0<br>1 1<br>2 2<br>3 3<br>4 4<br>5 5<br>6 6<br>7 7<br>8 8 |

| | | | |
|---|---|---|---|
| | | | 9 9<br>10 10 |
| 133 | [n_followup_q1c] | 1c. If positive, fill seroconversion form | dropdown, Required<br>0 0<br>1 1<br>2 2<br>3 3<br>4 4<br>5 5<br>6 6<br>7 7<br>8 8<br>9 9<br>10 10 |
| 134 | [n_followup_q2a] | Section Header: *FOR PREGNANT PATIENT ONLY: At the 1 month or 3 months or yearly follow up visits only: Review/repeat serum creatinine result*<br>2a. If serum creatinine >85umol/l, stop PrEP and repeat serum creatinine in one week. If still high, refer to Dr for more investigations | dropdown, Required<br>0 0<br>1 1<br>2 2<br>3 3<br>4 4<br>5 5<br>6 6<br>7 7<br>8 8<br>9 9<br>10 10 |
| 135 | [n_followup_q2b] | 2b. If creatinine< 85umol/l, continue PrEP | dropdown, Required<br>0 0<br>1 1<br>2 2<br>3 3<br>4 4<br>5 5<br>6 6<br>7 7<br>8 8<br>9 9<br>10 10 |

| | | | |
|---|---|---|---|
| 136 | [ n_followup_q3a ] | Section Header: *FOR BREASTFEEDING PATIENT ONLY: check creatinine (at baseline and annually) if >30 years or have diabetes/hypertension*<br>3a. If eGFR< 50mL/min, stop PrEP and recheck creatinine after one week, If still high, refer | dropdown, Required<br>0 0<br>1 1<br>2 2<br>3 3<br>4 4<br>5 5<br>6 6<br>7 7<br>8 8<br>9 9<br>10 10 |
| 137 | [ n_followup_q3b ] | 3b. If eGFR >50mL/min continue PrEP | dropdown, Required<br>0 0<br>1 1<br>2 2<br>3 3<br>4 4<br>5 5<br>6 6<br>7 7<br>8 8<br>9 9<br>10 10 |
| 138 | [ n_followup_q4a ] | Section Header: *FOR ALL PATIENTS: Check Hepatitis B sAg test result*<br>4a. If result positive, refer to doctor for further test AND continue PrEP | dropdown<br>0 0<br>1 1<br>2 2<br>3 3<br>4 4<br>5 5<br>6 6<br>7 7<br>8 8<br>9 9<br>10 10 |
| 139 | [ n_followup_q4b ] | 4b. If result negative, inform patient of result and continue PrEP | dropdown<br>0 0 |

| | | | |
|---|---|---|---|
| | | | 1 1<br>2 2<br>3 3<br>4 4<br>5 5<br>6 6<br>7 7<br>8 8<br>9 9<br>10 10 |
| 140 | [n_followup_q5a] | Section Header: *Assess daily PrEP adherence:*<br>5a. Ask questions and allow client to speak about her own adherence | dropdown<br>0 0<br>1 1<br>2 2<br>3 3<br>4 4<br>5 5<br>6 6<br>7 7<br>8 8<br>9 9<br>10 10 |
| 141 | [n_followup_q5b] | 5b. Assess her reported adherence over last three days (or longer if missed past 3 days) | dropdown<br>0 0<br>1 1<br>2 2<br>3 3<br>4 4<br>5 5<br>6 6<br>7 7<br>8 8<br>9 9<br>10 10 |
| 142 | [n_followup_q5c] | 5c. Be non-judgmental | dropdown<br>0 0<br>1 1<br>2 2 |

| | | | |
|---|---|---|---|
| | | | 3 3<br>4 4<br>5 5<br>6 6<br>7 7<br>8 8<br>9 9<br>10 10 |
| 143 | [n_followup_q5d] | 5d. Remind patient what to do if imperfect use of PrEP (use condoms for 7 days) | dropdown<br>0 0<br>1 1<br>2 2<br>3 3<br>4 4<br>5 5<br>6 6<br>7 7<br>8 8<br>9 9<br>10 10 |
| 144 | [n_followup_q6] | 6. Review if patient has side effects and counsel appropriately | dropdown, Required<br>0 0<br>1 1<br>2 2<br>3 3<br>4 4<br>5 5<br>6 6<br>7 7<br>8 8<br>9 9<br>10 10 |
| 145 | [n_followup_q7] | 7. Remind patients of symptoms of acute HIV and to come back if they present | dropdown, Required<br>0 0<br>1 1<br>2 2<br>3 3<br>4 4 |

| | | | |
|---|---|---|---|
| | | | 5 5<br>6 6<br>7 7<br>8 8<br>9 9<br>10 10 |
| 146 | [n_followup_q8] | 8. Prescribe 3 months of PrEP and advise on effective use of PrEP if needed | dropdown, Required<br>0 0<br>1 1<br>2 2<br>3 3<br>4 4<br>5 5<br>6 6<br>7 7<br>8 8<br>9 9<br>10 10 |
| 147 | [n_followup_q9] | 9. Give next appointment date and phone # of clinic if problems/concerns | dropdown, Required<br>0 0<br>1 1<br>2 2<br>3 3<br>4 4<br>5 5<br>6 6<br>7 7<br>8 8<br>9 9<br>10 10 |
| 148 | [n_followup_time] | Time spent with patient (minutes):<br>*Minutes* | text (number, Min: 0), Required |
| 149 | [n_followup_overall] | Overall rating (0-10): | dropdown, Required<br>0 0<br>1 1<br>2 2<br>3 3<br>4 4<br>5 5 |

| | | | |
|---|---|---|---|
| | | | 6 6<br>7 7<br>8 8<br>9 9<br>10 10 |
| 150 | [n_followup_feedback] | Feedback given to nurse: | notes, Required |
| 151 | [n_followup_comment] | Any other comments | notes |
| 152 | [n_followup_initials] | Capturer's initials | text, Required |
| 153 | [prep_followup_nurse_assessment_checklist_complete] | Section Header: *Form Status*<br>Complete? | dropdown<br>0 Incomplete<br>1 Unverified<br>2 Complete |
| **Instrument: PrEP offer and uptake - Pregnant women (prep_offer_and_uptake_pregnant_women)** | | | |
| 154 | [prep_date] | Date | text (date_dmy, Min: 2022-03-01) |
| 155 | [prep_attended] | 1. Head count: # pregnant women attended antenatal care<br><br>*Source: Clinic Registration Form* | text (number, Min: 0), Required |
| 156 | [prep_art] | # Known HIV positive women attending ANC | text, Required |
| 157 | [prep_hivtest] | 2. # pregnant women tested<br><br>*Source: Counsellor's log* | text (number, Min: 0), Required |
| 158 | [prep_exposed] | 3. # Pregnant tested HIV positive?<br><br>*Source: Counsellor's log* | text (number, Min: 0), Required |
| 159 | [prep_hivnegative] | 4. Of #1, How many are HIV negative women?<br><br>*Source: Clinic Registration Form* | text (number, Min: 0), Required |
| 160 | [prep_offer] | 5. Of #2, # women were offered PrEP<br><br>*Source: HIV counselor reports/ log books* | text (number, Min: 0), Required |
| 161 | [prep_initiate] | 6. Of #3, # women initiated PrEP<br><br>*Source: Nurse log* | text (number, Min: 0), Required |
| 162 | [prep_declined] | 7. Of #4, # women declined PrEP<br><br>*Source: Number of women offered PrEP - number of women who initiated PrEP* | calc, Required<br>Calculation: [prep_offer]-[prep_initiate] |
| 163 | [prep_comment] | Any other comments | notes |
| 164 | [prep_initials] | Capturer initials | text, Required |

| | | | |
|---|---|---|---|
| 165 | [prep_offer_and_uptake_pregnant_women_complete] | Section Header: *Form Status*<br>Complete? | dropdown<br>0 Incomplete<br>1 Unverified<br>2 Complete |
| **Instrument: PrEP offer and uptake - Breastfeeding/postpartum women (prep_offer_and_uptake_breastfeedingpostpartum_wome)** | | | |
| 166 | [prep_date_v2] | Date | text (date_dmy, Min: 2022-03-01) |
| 167 | [prep_attended_v2] | 1. Head count: # babies attending wellness/baby clinic (as proxy for breastfeeding/postpartum women)<br>*Source: Nurse's tally sheet/RMR/facility headcount* | text (number, Min: 0), Required |
| 168 | [prep_art_v2] | # HIV exposed (PMTCT) babies attending wellness clinic (as proxy for postpartum Known HIV positive)<br>*Source: PMTCT folders* | text, Required |
| 169 | [prep_hivtest_v2] | 2. # breastfeeding/postpartum women tested<br>*Source: Counsellor's log* | text (number, Min: 0), Required |
| 170 | [prep_exposed_v2] | 3. Of #1, # Breastfeeding/postpartum tested HIV positive?<br>*Source: RMR Counsellor's log/book* | text (number, Min: 0), Required |
| 171 | [prep_hivnegative_v2] | 4. Of #2, How many are HIV negative women?<br>*Source: Clinic Registration Form* | text (number, Min: 0), Required |
| 172 | [prep_offer_v2] | 5. Of #4, # women were offered PrEP<br>*Source: HIV counselor reports/ log books* | text (number, Min: 0), Required |
| 173 | [prep_initiate_v2] | 6. Of #5, # women initiated PrEP<br>*Source: Nurse log* | text (number, Min: 0), Required |
| 174 | [prep_declined_v2] | 7. Of #6, # women declined PrEP<br>*Source: Number of women offered PrEP - number of women who initiated PrEP* | calc, Required<br>Calculation: [prep_offer_v2]-[prep_initiate_v2] |
| 175 | [prep_comment_v2] | Any other comments | notes |
| 176 | [prep_initials_v2] | Capturer initials | text, Required |
| 177 | [prep_offer_and_uptake_breastfeedingpostpartum_wome_complete] | Section Header: *Form Status*<br>Complete? | dropdown<br>0 Incomplete<br>1 Unverified<br>2 Complete |
| **Instrument: PrEP Continuation Report Form (prep_continuation_report_form)** | | | |
| 178 | [refill_date] | Date | text (date_dmy, Min: 2022-03-01), Required |
| 179 | [refill_pregnant] | Section Header: *Targets: >60% of women returned for a 1-month refill of PrEP prescription & we report on data* | text, Required |

| | | | |
|---|---|---|---|
| | | *correctly >50% of women returned for a 3-month and 6-month refill of PrEP prescription & we report on data correctly*<br>1. # PrEP refills among pregnant women in antenatal care<br>*Weekly total* | |
| 180 | [refill_postnatal] | 2. # PrEP refills among postnatal women in antenatal care<br>*Weekly total* | text, Required |
| 181 | [refill_comment] | Any other comments | notes |
| 182 | [refill_initials] | Capturer's initials | text (alpha_only), Required |
| 183 | [prep_continuation_report_form_complete] | Section Header: *Form Status*<br>Complete? | dropdown<br>0 Incomplete<br>1 Unverified<br>2 Complete |

**Instrument: Breastfeeding pilot (breastfeeding_pilot)** **[collapsed]**
