# Supplementary material for: Integration of HIV pre-exposure prophylaxis (PrEP) services for pregnant and breastfeeding women in eight primary care clinics: results of an implementation science study
Source: BMC Glob Public Health. 2024 Aug 26;2:57. doi: 10.1186/s44263-024-00089-8 (PMC11622949; doi:10.1186/s44263-024-00089-8)
Supplement: Supplementary file 4 — Additional file 4. Adoption of PrEP guidelines by healthcare providers measured by post-test questionnaire results and mentoring score [file 44263_2024_89_MOESM4_ESM.docx]

**Table S1. Adoption of PrEP guidelines by healthcare providers measured by post-test questionnaire results and mentoring score**

|  | **Overall**  **(N = 224)** | **Non-ART trained / midwives**  **(n = 75, 33%)** | **ART-trained nurses / midwives**  **(n = 52, 23%)** | **Counsellor**  **(n = 37, 17%)** | **Healthcare assistant**  **(n = 33, 15%)** | **Admin clerk**  **(n = 16, 7%)** | **Clinic manager**  **(n = 8, 4%)** | **Other HCW***  **(n = 3, 1%)** |
| --- | --- | --- | --- | --- | --- | --- | --- | --- |
| **Pre-training test score as %, median (IQR)** | 70 (50, 80) | 70 (60, 80) | 70 (60, 80) | 60 (50, 70) | 50 (40, 60) | 50 (40, 60) | 60 (48, 70) | 60 (55, 75) |
| **Post-training test score as %, median (IQR)** | 80 (70, 90) | 80 (80, 90) | 90 (80, 90) | 80 (70, 80) | 70 (50, 80) | 60 (50, 70) | 80 (70, 90) | 80 (70, 90) |
| **Number with post training test ≥80%** | 129 (58%) | 54 (72%) | 42 (80%) | 14 (38%) | 11 (33%) | 2 (13%) | 5 (63%) | 1 (33%) |
| **% change in pre to post-test**  **(t-test)** | 31.15% | 21.29%  (p < 0.001) | 30.59%  (p < 0.001) | 33.82%  (p < 0.001) | 60.70%  (p = 0.004) | 9.15%  (p = 0.105) | 45.04%  (p = 0.004) | 15.56%  (p 0.633) |
| **Number mentored** | 60 | 20 | 20 | 20 | - | - | - | - |
| **Initial mentoring score ≥ 8, n (%)** | 34 of 60 (57%) | 13 of 20 (65%) | 13 of 20 (65%) | 8 of 20 (40%) | - | - | - | - |
| **Initial mentoring score as %, median (IQR)**** | 8 (7, 8.6) | 8  (7, 9) | 8  (7.1, 8) | 7.7 (6, 8.9) | - | - | - | - |
| **Number of staff with final mentoring score ≥8** | 45 of 60 (75%) | 16 of 20 (80%) | 16 of 20 (80%) | 13 of 20 (65%) |  |  |  |  |
| **Final mentoring score, median (IQR)**** | 8 (8, 9) | 8 (8, 9) | 8 (8, 9) | 8 (6, 9) | - | - | - | - |
| * Other HCWs include 2 physicians and 1 pharmacist  **in cases where multiple assessments were taken for one person on the same day, their average score was used | | | | | | | | |
