# Supplementary material for: Integration of HIV pre-exposure prophylaxis (PrEP) services for pregnant and breastfeeding women in eight primary care clinics: results of an implementation science study
Source: BMC Glob Public Health. 2024 Aug 26;2:57. doi: 10.1186/s44263-024-00089-8 (PMC11622949; doi:10.1186/s44263-024-00089-8)
Supplement: Supplementary file 5 — Additional file 5. Demographics of healthcare providers who completing the PrEP training and mentorship (April 2022- January 2023) [file 44263_2024_89_MOESM5_ESM.docx]

**Table S2. Demographics of healthcare providers who completing the PrEP training and mentorship (April 2022- January 2023)**

|  | **Overall** | **Nurses** | **Midwives** | **Counsellor** | **Health assistants** | **Admin clerk** | **Clinic manager** | **Other HCW*** |
| --- | --- | --- | --- | --- | --- | --- | --- | --- |
|  | N (%) | n (%) | n (%) | n (%) | n (%) | n (%) | n (%) | n (%) |
| **Total** | **224** | **108 (48%)** | **19 (8%)** | **37 (17%)** | **33 (15%)** | **16 (7%)** | **8 (4%)** | **3 (1%)** |
| Post-secondary school qualifications | 177 (79%) | 108 (100%) | 19 (100%) | 3 (8%) | 33 (100%) | 3 (19%) | 8 (100%) | 3 (100%) |
| Years of experience (median, IQR) | 7 (4-11) | 5 (3-10) | 10 (7-15) | 9 (7- 10) | 10 (5-13) | 7 (5- 11) | 10  (7-18) | 8 (6-9) |
| Worked with PrEP before | 27 (12%) | 18 (17%) | 1 (5.6%) | 6 (17%) | 0 (0%) | 0 (0%) | 2 (29%) | 0 (0%) |
| DOH PrEP training done (online) | 39 (18%) | 20 (19%) | 2 (11%) | 14 (42%) | 1 (3%) | 0 (0%) | 2 (29%) | 0 (0%) |
| ART-trained | 52 (23%) | 45 (42%) | 7 (37%) | - | - | - | - | - |
| *** Other HCWs include 2 physicians and 1 pharmacist | | | | | | | | |
